# Near-zero stiffness accelerometer with buckling of tunable electrothermal microbeams

**DOI:** 10.1038/s41378-024-00657-w

**Published:** 2024-03-22

**Authors:** Hussein Hussein, Chen Wang, Rui Amendoeira Esteves, Michael Kraft, Hossein Fariborzi

**Affiliations:** 1 Department of Mechanical Engineering, MSFEA, American University of Beirut, Beirut, 1107 2020 Lebanon; 2 King Abdullah University of Science and Technology, Thuwal, 23955-6900 Saudi Arabia; 3 ESAT-MNS, University of Leuven, 3001 Leuven, Belgium

**Keywords:** Engineering, Physics

## Abstract

Pre-shaped microbeams, curved or inclined, are widely used in MEMS for their interesting stiffness properties. These mechanisms allow a wide range of positive and negative stiffness tuning in their direction of motion. A mechanism of pre-shaped beams with opposite curvature, connected in a parallel configuration, can be electrothermally tuned to reach a near-zero or negative stiffness behavior at the as-fabricated position. The simple structure helps incorporate the tunable spring mechanism in different designs for accelerometers, even with different transduction technologies. The sensitivity of the accelerometer can be considerably increased or tuned for different applications by electrothermally changing the stiffness of the spring mechanism. Opposite inclined beams are implemented in a capacitive micromachined accelerometer. The measurements on fabricated prototypes showed more than 55 times gain in sensitivity compared to their initial sensitivity. The experiments showed promising results in enhancing the resolution of acceleration sensing and the potential to reach unprecedent performance in micromachined accelerometers.

## Introduction

Micromachined accelerometers are increasingly implemented in mobile and testing devices to measure motion, shocks, and vibrations. They find applications in a wide range of scenarios, including inertial navigation for land^1^, water^2^, aerial^3^, and space^4^ vehicles, as well as seismology^5^, oil exploration^6^, smartphones^7^, wearable devices^8^, health monitoring^9^, vibration monitoring^10^, and shock sensing^11^. These accelerometers come in various designs, offering different sensitivity, resolution, dynamic range, and frequency response characteristics to suit different applications. Tunable accelerometers, in particular, can perform measurements in several modes of operation, adapting to a wide array of applications, each with varying requirements.

Enhancing the measurement performance of accelerometers holds great potential, particularly in fields such as seismology, oil exploration, gravimetry, and high-precision navigation. Improved resolution and low noise levels in acceleration measurements would enhance the functionality and detection capability in these applications. To achieve high-resolution measurements, several approaches have been explored for micromachined accelerometers^12^. These approaches include lowering spring stiffness^13^, increasing proof mass size^14^, enhancing the quality factor^15^, designing low-noise readout circuits^16^, and utilizing high-sensitivity transducers^17^.

Recent innovations have introduced a new approach to improving the resolution of MEMS accelerometers based on the anti-spring effect, resulting in designs with near-zero stiffness. These “anti-spring" mechanisms fall into two categories. The first category exhibits near-zero stiffness far from the as-fabricated configuration, necessitating pre-loading after fabrication. The sensitivity curve remains constant over a range of displacement, and pre-loading can be achieved through passive means, such as gravity^13,18^, or through active methods using built-in actuators^19,20^. The achievable stiffness limit with these mechanisms is constrained by fabrication tolerances, which significantly affect the sensitivity curves.

The second category achieves near-zero stiffness at the as-fabricated position of the proof mass by tuning the complete stiffness-displacement behavior of the spring mechanism. This category outperforms the first category in sensitivity to fabrication imperfections, as the characteristic stiffness curve can be adjusted to compensate for tolerances. Guo et al.^21^ proposed tuning the stiffness behavior using opposite electrostatic forces on the proof mass. However, sensitivity gain through electrostatic tuning is limited by the pull-in phenomenon. Duan et al.^22^ suggested longitudinally compressing thin beams with electrothermal actuators to induce buckling and alter their lateral stiffness behavior. The bulky actuators added to the hanging mobile parts in the accelerometer result in a more fragile and bulky structure for the accelerometer.

**Fig. 1:**
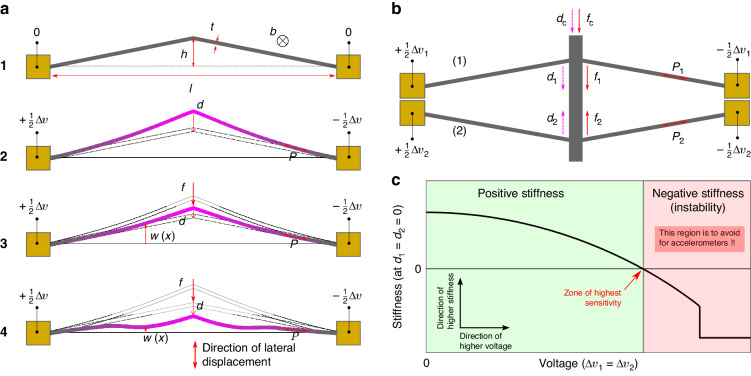
The tunable stiffness mechanism and its principle. **a** Schematic of an inclined beam V-shaped actuator. **a1** Dimensions of the beam in the as-fabricated configuration. **a2** Free displacement after applying a potential difference to the actuator boundaries. **a3** Lateral loading on the actuator. **a4** Buckling (third mode) when the actuator reaches a high level of axial compression. **b** Mechanism of opposite inclined beams with tunable lateral stiffness. **c** A typical stiffness curve for the tunable stiffness mechanism showing a reduction in the stiffness after electro-heating the opposite inclined beams. High sensitivity can be reached with positive near zero stiffness spring. While negative stiffness has potential advantages for different applications, accelerometers cannot have negative stiffness in their springs as they would have two possible outputs for the same acceleration

This paper introduces a tunable and high-sensitivity accelerometer based on a tunable-stiffness spring mechanism. The tunable spring mechanism consists of a symmetric set of pre-shaped beams with identical dimensions but opposite curvature^23^, as illustrated in Fig. 1b. The initial stiffness of the spring in the sensitive direction can be reduced by electrothermally heating its structure until it reaches near-zero stiffness behavior, as depicted in Fig. 1c. Thus, the accelerometer’s sensitivity is tuned by electrothermally adjusting the spring mechanism’s stiffness.

The pre-shaped beams exhibit nonlinear and non-symmetrical stiffness behavior in their lateral motion range. Electrothermally compressing the pre-shaped beams reduces their lateral stiffness at the as-fabricated position. Connecting opposite pre-shaped beams, as in the proposed tunable stiffness spring, equalizes the actuating forces between the two lateral sides. Consequently, heating the beams in the opposite beam mechanism reduces the lateral stiffness without displacing the beams from their initial position. The reduction in stiffness in the lateral direction occurs due to the high axial forces applied to the beams due to electroheating and just before the buckling at the third mode, which occurs to limit the increase in axial forces. Based on this principle, the proposed tunable stiffness spring falls into the second category of anti-spring mechanisms.

As a proof of concept, the tunable spring mechanism with inclined beams is implemented in a capacitive accelerometer, with demonstrated performance using a mature fabrication process^24,25^. The accelerometer’s design varies only in the spring structure dimensions. An interface circuit enables the electrothermal heating of the spring. The experiments demonstrate efficient sensitivity tuning for the fabricated prototypes, achieving a considerable sensitivity increase in certain prototypes by lowering their stiffness to a very low positive value. Compared to the accelerometer design in^22^, the proposed mechanism in this work features a more compact and simpler structure, eliminating the need for additional actuators to induce buckling.

The novel tunable spring mechanism offers several advantages. Firstly, its simplicity facilitates implementation in various MEMS accelerometer designs, making it versatile for enhancing measurement accuracy in high-performance accelerometers. It allows existing accelerometer designs to tune their sensitivity by simply replacing their springs with the proposed spring mechanism. Secondly, there is potential for further boosting sensitivity beyond the reported limits by improving the electronic interface and optimizing measurement conditions. Thirdly, the tunable spring mechanism achieves extremely low stiffness while maintaining low cross-axis sensitivity and high robustness. Additionally, it overcomes the limitations of combining positive and negative stiffness mechanisms, as it can achieve lower stiffness limits without being affected by fabrication tolerances. Lastly, the tunability aspect makes it suitable for various applications with different performance requirements.

The working principle of the tunable spring mechanism, the governing equations, and the impact on the accelerometer’s performance are theoretically investigated and derived in the first part of the “Results" Section. The experimental measurements on the fabricated prototypes are demonstrated afterward. The proposed spring mechanism has the potential to push the performance of micromachined accelerometers to unprecedented limits, whether through optimized spring beam dimensions or improved measurement conditions, as discussed in the “Discussion" section. The “Methods" section includes a simulation to confirm electrical compatibility with the spring mechanism, details on the fabrication process, and an explanation of the experimental setup.

## Results

### Theory and design

#### Inclined beams V-shaped actuators

Positive stiffness behavior is a property of deformable structures or devices in their stable configuration. Bistable mechanisms are known to have two stable configurations with negative stiffness behavior in the transition zone^26^. Due to their simple structure, compactness, and interesting properties, pre-shaped beams (curved, inclined, arc-shaped, etc.) are widely used in MEMS as compliant bistable mechanisms. Several mechanisms with interesting behavior at small and large scales have been developed, relying on the special stiffness behavior of pre-shaped beams^27^, including negative stiffness mechanisms^28^, zero-force mechanisms^29^, tunable stiffness mechanisms^30^, large-stroke mechanisms^31^, metamaterials^32^, safe manipulation^33^, accurate positioning^34^, and high sensitivity sensors^21^.

On the other hand, inclined or V-shaped beams are widely used in MEMS as electrothermal actuators due to their simple structure, simple control, and large stroke and force capabilities compared to other actuators on the small scale. The deflection of the V-shaped actuator is controlled by passing electrical current through the beam structure. The beam heating due to the Joule effect expands the beam length and increases the axial force in the beam structure. This results in an amplified lateral displacement at the mid-length of the actuator^35,36^. Figure 1a shows a schematic clarifying the main dimensions for the inclined beam actuator (a1), the deflection after electro-heating the actuator (a2), after applying lateral loading (a3), and the buckling at the third mode that occurs when the beam length is highly compressed (a4).

The inclined beam has a length *l*, thickness *t*, mid-length height *h*, and out-of-plane width *b*. The beam is electro-heated by applying a potential difference Δ*v* to its boundaries. This adds an axial force *P* along the beam length and induces a lateral deflection *d* at the mid-length of the beam. Applying a lateral loading or snapping force *f* changes the deflection and axial force levels. The snap-through behavior of the inclined beam is governed by the following two analytical equations, which relate the axial and snapping forces, beam deflection, and electrothermal input^35,36^:

1a $$F=4{N}^{2}-\frac{{{\Delta }}}{{{{\Lambda }}}_{e}}$$

1b $$\frac{{{{\Lambda }}}_{a}}{{{{\Lambda }}}_{e}^{2}}{{{\Delta }}}^{2}-4{{\Delta }}+\frac{{N}^{2}-{V}^{2}}{12{Q}^{2}}=0$$

where Δ, *N*, and *F* are the normalized deflection, axial force, and snapping force, respectively. *Q* is the height-to-thickness ratio, *E* is the Young’s modulus, and *I* is the cross-section quadratic moment.

2 $$\begin{array}{c}Q=\displaystyle\frac{h}{t};{{\Delta }}=\frac{d}{h};N=\sqrt{\frac{P{l}^{2}}{EI}};F=\frac{f{l}^{3}}{EIh};I=\frac{b{t}^{3}}{12};\end{array}$$

The coefficients Λ_*a*_ and Λ_*e*_ are dependent on *N*:

3a $${{{\Lambda }}}_{a}(N)=\frac{3}{16{N}^{4}}\left(1-\frac{\tan \frac{N}{4}}{\frac{N}{4}}+\frac{{\tan }^{2}\frac{N}{4}}{3}\right)$$

3b $${{{\Lambda }}}_{e}(N)=\frac{1}{{N}^{3}}\left(\frac{N}{4}-\tan \frac{N}{4}\right)$$

The parameter *V* in (1) is the normalized axial force due to electrothermal heating:

4 $${V}^{2}=12\frac{l}{{t}^{2}}{{\Delta }}{l}_{h}$$

where Δ*l*_*h*_ is the length expansion due to heating.

Considering a simple electrothermal model with neglected convection and radiation and constant material properties^36^, *V* is calculated as follows:

5 $${V}^{2}=\frac{{{\Delta }}{v}^{2}\alpha {l}^{2}}{{\rho }_{0}{K}_{p}{t}^{2}}$$

where Δ*v* is the potential difference applied to the beam boundaries, *α* is the thermal expansion coefficient, *K*_*p*_ is the conduction coefficient, and *ρ*_0_ is the electrical resistivity.

**Fig. 2:**
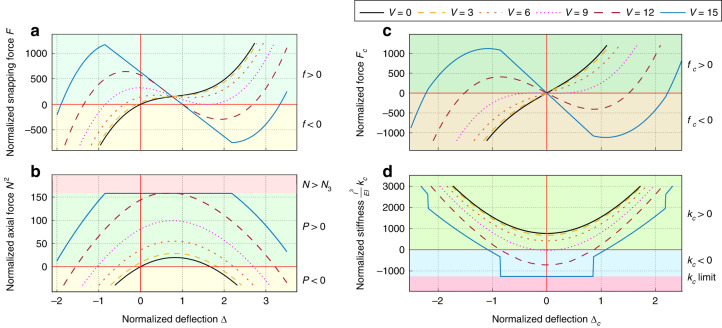
Characteristic curves from the analytical modeling. **a** Normalized snapping force curve for one inclined beam. **b** Normalized axial force curve for the inclined beam. **c** Normalized force curve for a tunable stiffness mechanism similar to the mechanism shown in Fig. 1b. **d** Normalized stiffness curve for the tunable stiffness mechanism. The curves are shown considering *Q* = 1 and for different values of *V* as depicted in the legend

Mechanically, the main structural parameter affecting the stiffness of the inclined beam actuator is the axial force, which increases to a maximum in the negative stiffness zone. Fig. 2a, b show the variation of the normalized snapping force-deflection (*F* − Δ) and axial force-deflection (*N* − Δ) curves, respectively, at different values of *V* for the case of a height-to-thickness ratio *Q* = 1. Depending on the dimensions and electrothermal input, the inclined beam can have one stable position on the first side of buckling (cases of *V* ≤ 6 in Fig. 2a), or two stable positions on both sides of buckling (cases of *V* ≥ 9).

At the free stable position (where *f* = 0 and ∂*f*/∂*d* > 0), *N* has a positive value between [0, *N*_3_ = 4*π*]. *N* increases to a maximum in the middle snapping zone between the two sides of buckling (around Δ = 1 or *d* = *h*), where the beam length is compressed. A bifurcation of solution occurs when *N* reaches *N*_3_, and the third mode of buckling is involved in the beam shape to relax the beam compression and avoid exceeding *N* = *N*_3_. Mathematically, the bifurcation of the solution occurs in the range of deflection where *N* calculated from (1) is higher than *N*_3_. In this range, *N* is set equal to *N*_3_, and the relation between *F* and Δ becomes linear with a negative stiffness behavior.

6 $$F=64{\pi }^{2}\left(1-{{\Delta }}\right)$$

On the other side, when the beam is laterally moved far from the middle zone of buckling, the beam compression is relaxed until reaching the tension zone (*P* < 0). In the case of axial tension, *N* would have an imaginary value ($N=i\sqrt{-P{l}^{2}/EI}$), and equations (1) and (3) remain applicable.

The snapping force expressions (1) and (6) have been proven to be in excellent agreement with various finite element simulations and experimental measurements under different conditions^26,36–38^. This work relies on these expressions to further analyze the stiffness behavior of a tunable spring system consisting of inclined beams. The stiffness of the inclined beam in the lateral direction is calculated from the force-deflection relationship:

7 $$k=\frac{\partial f}{\partial d}=\frac{EI}{{l}^{3}}\frac{\partial F}{\partial {{\Delta }}}$$

The derivative ∂*F*/∂Δ is calculated from (1) and (6):

8 $$\frac{\partial F}{\partial {{\Delta }}}=\left\{\begin{array}{ll}\frac{-6\frac{{Q}^{2}}{N}\frac{{{{\Lambda }}}_{a}^{{\prime} }}{{{{\Lambda }}}_{e}^{3}}{{{\Delta }}}^{2}+192{Q}^{2}\left(1-\frac{{{{\Lambda }}}_{a}}{{{{\Lambda }}}_{e}^{2}}{{\Delta }}\right)-\frac{1}{{{{\Lambda }}}_{e}}}{6{Q}^{2}\left(\frac{1}{N}\frac{{{{\Lambda }}}_{a}^{{\prime} }}{{{{\Lambda }}}_{e}^{2}}+\frac{8{{{\Lambda }}}_{a}^{2}}{{{{\Lambda }}}_{e}^{3}}\right){{{\Delta }}}^{2}+1}&N \, < \, {N}_{3}\\ -64{\pi }^{2}&N={N}_{3}\end{array}\right.$$

where ${{{\Lambda }}}_{a}^{{\prime} }$ is the derivative ∂Λ_*a*_/∂*N*:

9 $${{{\Lambda }}}_{a}^{{\prime} }=-\frac{1}{2{N}^{5}}+\frac{15\tan \left(\frac{N}{4}\right)}{4{N}^{6}}+\frac{N\tan \left(\frac{N}{4}\right)-14}{32{N}^{5}{\cos }^{2}\left(\frac{N}{4}\right)}$$

#### Opposite beams tunable stiffness mechanism

A mechanism consisting of opposite pre-shaped beams, as depicted in Fig. 1b, exhibits tunable stiffness behavior controlled by electrical input^23^. The opposite beams in this mechanism have the same dimensions and are connected in a parallel configuration to a rigid shuttle at their mid-length.

The force, deflection, and stiffness parameters for the top beam (*f*_1_, *d*_1_, *P*_1_, *k*_1_) and bottom beam (*f*_2_, *d*_2_, *P*_2_, *k*_2_) are calculated separately using the equations (1)–(9) governing the snap-through of a single beam. The resulting force, deflection, and stiffness parameters for the opposite beams mechanism depicted in Fig. 1b are calculated as follows:

10a $${f}_{c}={f}_{1}-{f}_{2}$$

10b $${d}_{c}={d}_{1}=-{d}_{2}$$

10c $${k}_{c}={k}_{1}-{k}_{2}$$

In addition to the geometrical similarity in the dimensions of the opposite beams, we consider that the same electrothermal input is applied to all the beams:

11a $${{\Delta }}{v}_{c}={{\Delta }}{v}_{1}={{\Delta }}{v}_{2}$$

11b $${V}_{c}={V}_{1}={V}_{2}$$

The symmetry in the geometry and similarity in the electrothermal input result in a symmetrical snap-through behavior for the combined beams (*f*_*c*_( − *d*_*c*_) = − *f*_*c*_(*d*_*c*_)). In these conditions, the resulting force at the initial position is equal to zero (*f*_*c*_ = 0 for *d*_*c*_ = 0). Figure 2c, d show the snapping force and sensitivity curves, respectively, for the mechanism of opposite inclined beams when varying the electrothermal input, considering a height-to-thickness ratio *Q* = 1.

The lateral stiffness decreases with the applied electricity, as can be concluded from Fig. 2d. The variation in stiffness is more significant when *V*_*c*_ is closer to *N*_3_. Due to buckling at the third mode, the stiffness becomes constant and negative around the initial position when *V*_*c*_ exceeds *N*_3_.

At the initial position, both *N*_1_ and *N*_2_ are equal to *V*_*c*_ (1b), and the sensitivity expression reduces to the following:

12 $${k}_{{c}_{0}}=2\frac{EI}{{l}^{3}}\left\{\begin{array}{ll}192{Q}^{2}-\frac{1}{{{{\Lambda }}}_{e}({V}_{c})}&{V}_{c} \,<\, {N}_{3}\\ -64{\pi }^{2}&{V}_{c}\ge {N}_{3}\end{array}\right.$$

**Fig. 3:**
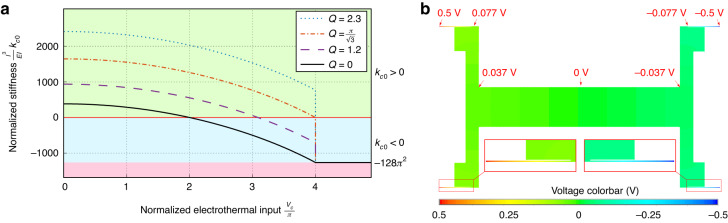
Stiffness behavior and electrical potential distribution. **a** Stiffness curves for the tunable stiffness mechanism calculated with respect to the normalized electrothermal input *V*_*c*_ for different values of the height-to-thickness ratio *Q*. **b** Electrical Potential across the proof mass simulated on Ansys considering 1 V potential difference between the two sides of the spring mechanism

Figure 3a shows the variation of the stiffness at the initial position with respect to the normalized electrothermal input *V*_*c*_ for different values of the height-to-thickness ratio *Q*.

The opposite beams mechanism has a single stable position as long as the stiffness at the initial position is positive. Once the stiffness at the initial position becomes negative, the initial position becomes unstable, and two stable positions are created on the two lateral sides. The distance between the two stable positions increases with further increase in the electrical input.

The initial position stiffness curves in Fig. 3a show a discontinuity at *V*_*c*_ = *N*_3_, where they maintain a fixed negative value for *V*_*c*_ > *N*_3_. In the range *V*_*c*_ < *N*_3_, the initial position stiffness reaches a zero level for a value of *V*_*c*_ = *V*_0_ between 2*π* and 4*π* for $Q\le \pi /\sqrt{3}$. The initial position cannot have a zero-stiffness level for $Q \,> \,\pi /\sqrt{3}$. Reaching positive near-zero stiffness is important for the accelerometer application as it leads to a significant increase in sensitivity.

#### Tunable stiffness spring in the accelerometer

Accelerometers work on the principle of a mass on a spring. Once accelerated, an inertia force applies to the mass and induces a deflection of the spring. The deflection can be measured using various transduction technologies, such as capacitive^25^, piezoelectric^39^, optical^40^, thermal^41^, and tunnel effect^42^ transducers. Capacitive accelerometers are the most widespread due to their high sensitivity, high selectivity, low noise, static measurement, small footprint, low cost, established fabrication process, and the possibility of integration with standard electronics.

**Fig. 4:**
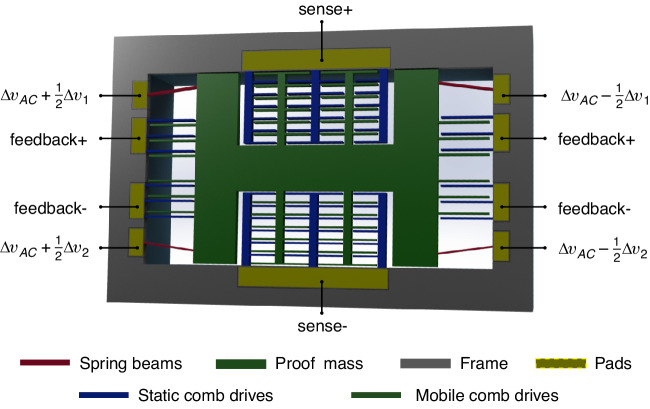
Schematic of an accelerometer with a tunable stiffness spring. Micromachined capacitive accelerometer with opposite inclined beams implemented as the spring mechanism

Figure 4 shows a typical configuration for a capacitive accelerometer. This configuration is representative of the accelerometer reported in^24,25^. The accelerometer consists of a proof mass suspended by compliant beams anchored to a fixed frame. The proof mass has a mass *m* proportional to its size. The compliant anchored beams are considered as the spring mechanism for the accelerometer. These beams are replaced by the opposite inclined beams mechanism reported in this work with an effective spring constant (stiffness) *k*_*c*_. The configuration in Fig. 4 is for a single-axis accelerometer as the springs have a relatively low stiffness in one direction (sensing direction), and the sensing comb drives are directed to measure the displacement in the sensing direction.

Compared to the configuration in Fig. 1b, the proof mass is considered as the shuttle of the opposite inclined beams mechanism in the accelerometer configuration of Fig. 4. The proof mass tends to resist the acceleration due to inertia, creating a force that deforms the spring mechanism and moves the proof mass in the sensing direction. Capacitive comb drives in a differential configuration are implemented to measure the proof mass displacement. Additional feedback comb drives are added for closed-loop operation.

The differential capacitance measurement is proportional to the proof mass displacement. The latter is proportional to the applied acceleration component in the sensing direction within a bandwidth covering static measurement and limited by the resonant frequency. The sensitivity decreases after the resonance peak. The resonant frequency depends on the mass and spring stiffness.

13 $${\omega }_{n}=\sqrt{\frac{{k}_{c}}{m}}$$

In the effective measurement bandwidth (acceleration angular frequency *ω* < < *ω*_*n*_), the mechanical sensitivity of the accelerometer or the displacement-to-acceleration ratio is inversely proportional to the square of the resonant frequency.

14 $${S}_{m}\approx \frac{1}{{\omega }_{n}^{2}}=\frac{m}{{k}_{c}}$$

The sensitivity is inversely proportional to the stiffness. Thus, tuning the stiffness to reach a near-zero positive value would highly increase the sensitivity of the accelerometer. Tuning the stiffness to a near-zero level should be highly accurate to prevent reaching a negative value for the stiffness, where the initial position for the proof mass becomes unstable. In that case, the system will have two stable positions, and thus two possible measurements for the same acceleration input. In this context, targeting the buckling at the third mode for the spring beams should be avoided as it indicates negative stiffness and instability of the proof mass position. However, amplifying the sensitivity of the accelerometer can be achieved by aiming for positive near-zero stiffness, which occurs just before reaching buckling.

On the other hand, the measurement resolution of the accelerometer is limited by the noise of the system. The mechanical and electronic noises must be minimized to achieve a resolution below microgravity. The total noise in the acceleration domain *N*_*t*,*a*_ is calculated as follows:

15 $${N}_{t,a}=\sqrt{{N}_{m,a}^{2}+\frac{{N}_{e,c}^{2}}{{S}_{cm}^{2}}}$$

where *N*_*m*,*a*_ is the mechanical noise, *N*_*e*,*c*_ is the electrical noise of the capacitive readout circuit, and *S*_*cm*_ is the capacitance-to-acceleration sensitivity of the accelerometer. Note that *S*_*cm*_ = *S*_*s*_*S*_*m*_, where *S*_*s*_ is the capacitance-to-displacement sensitivity of the sensing comb-drives.

*N*_*t*,*a*_ represents the minimum detectable capacitance variation. The resolution of the readout circuit is characterized by *N*_*e*,*c*_, which represents the minimum capacitance variation that the readout circuit can detect. Thus, decreasing the stiffness to near-zero in the tunable stiffness spring increases the mechanical sensitivity and allows for the measurement of smaller variations in acceleration using the same readout circuit.

The dynamic of the proof mass is damped, mainly due to the squeeze film damping between the comb drives and surrounding fluids, with an effective damping coefficient *b*. The dominant source of mechanical noise for relatively small-sized micromachined devices is molecular Brownian motion noise, which increases with damping.

The mechanical noise *N*_*m*,*a*_ is calculated as follows^43^:

16 $${N}_{m,a}=\frac{\sqrt{4{k}_{B}Tb{{\Delta }}f}}{m}$$

where *k*_*B*_ is the Boltzmann constant, *T* is the temperature, and Δ*f* is the bandwidth.

The quality factor of the accelerometer is inversely proportional to the damping coefficient. The damping due to Brownian motion is proportional to the viscosity of the fluid surrounding the accelerometer structure. Viscosity depends on fluid pressure and can be reduced by several orders of magnitude with vacuum packaging. Increasing the quality factor with vacuum packaging is an efficient solution to improve the resolution of accelerometers^44^. However, at low pressure, the system becomes underdamped with a damping ratio *ζ* < 1. Underdamped behavior is undesirable for accelerometer operation as the system oscillates with a large response time. Therefore, high-quality factor accelerometers usually require closed-loop control to enhance their stability and response time. Note that closed-loop measurement adds additional noise from the feedback circuitry to the total system noise. The damping ratio *ζ* is calculated as follows:

17 $$\zeta =\frac{b}{2\sqrt{m{k}_{c}}}$$

As *ζ* increases for low stiffness (17), reducing the stiffness to near-zero using the tunable stiffness mechanism helps enhance the stability and response time of the accelerometer in a vacuum. In this sense, mechanical noise can be reduced to a very low level in a vacuum without the need for closed-loop measurements. Moreover, increasing the quality factor, which is inversely proportional to *ζ*, is highly beneficial for other devices and applications relying on resonance.

### Experiments on fabricated prototypes

**Fig. 5:**
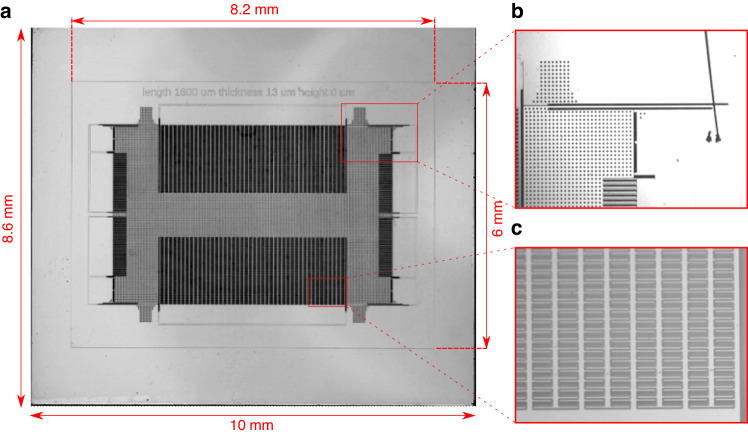
The accelerometer die. **a** Microscopic photo of a fabricated accelerometer die after release. **b** Zoom on one corner of the tunable spring mechanism. **c** Zoom on the sensing comb drives

The simple structure of the opposite beams mechanism enables its implementation in different accelerometer designs and with various types of transducers. As a proof of concept, we chose to implement the opposite-beams tunable spring mechanism in a capacitive accelerometer, which has demonstrated performance and a mature fabrication process^24,25^. During the design phase, we only replaced the spring beams on the four corners of the accelerometer with the opposite beams mechanism, as depicted in Fig. 4. The layout for the other parts of the accelerometer, as designed in^24,25^, was mostly retained. The beams in the spring mechanism have an inclined shape and were implemented in different dimensions, as illustrated in Table 1. Figure 5a shows a microscopic photo of a fabricated accelerometer with external dimensions of 10 mm ⋅ 8.6 mm for the support frame in the handle layer and 8.2 mm ⋅ 6 mm for the frame in the device layer. Figure 5b, c show zooms on the spring and sensing comb drives areas in the accelerometer die.

**Table 1:** Dimensions for the opposite inclined beams springs implemented in the fabricated accelerometer

	*b* (*μm*)	*t* (*μm*)	*h* (*μm*)	*l* (*mm*)
Spring 1	25	13	0	1.6
Spring 2	25	13	7	1.6
Spring 3	25	13	13	1.6
Spring 4	25	16	0	3
Spring 5	25	16	8	3

**Fig. 6:**
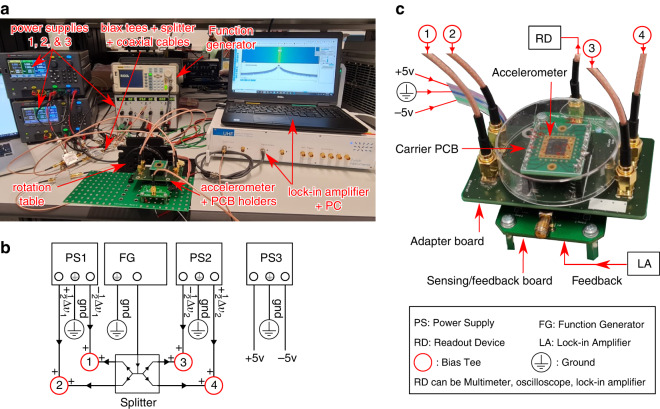
Experimental setup. **a** A photo for the experimental setup. **b** Powering circuit. **c** Wiring for the experimental measurements

The experiments were conducted on five sensor designs (with spring mechanisms 1-5) to demonstrate the ability to tune the stiffness and sensitivity in the fabricated prototypes. The experimental setup is shown in Fig. 6a and the wiring for the experimental measurements is illustrated in Fig. 6b, c.

**Fig. 7:**
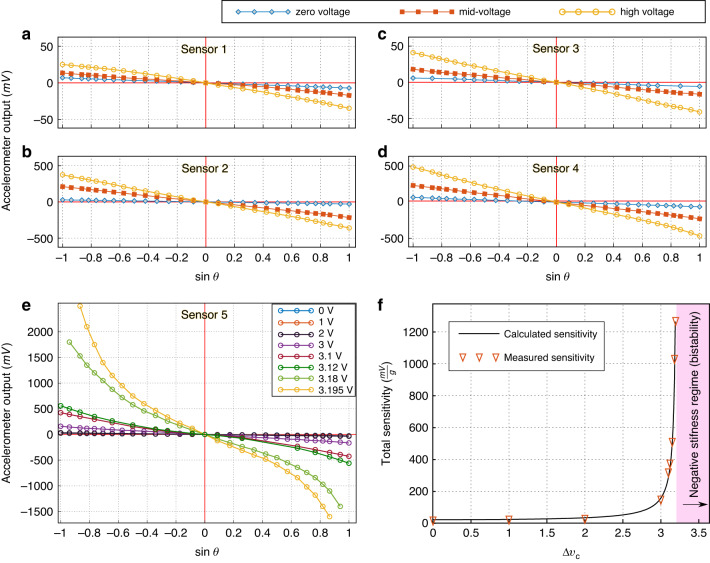
Sensitivity measurement. **a**–**e** Measured sensitivity for the sensor designs 1 to 5, respectively. The sensitivity curves of the first four designs are shown for three levels of applied voltages. Additional curves are shown for sensor 5, showing large improvement in sensitivity. **f** Comparison between calculated and measured total sensitivity for sensor 5

In the sensitivity measurements, the accelerometer with readout electronics was mounted on a rotation table (Thorlabs XRR1/M), capable of making a full rotation around its axis with a resolution of 5.78 mrad per revolution. The sensing axes of the accelerometer were aligned with the rotation direction. Thus, rotating the stage by an angle *θ* would exert an inertia force proportional to $g\sin \theta$ on the accelerometer in the sensing direction, where *g* is the acceleration due to gravity. Fig. 7a–e shows the sensitivity curves measured for accelerometer designs 1 to 5, respectively. These curves illustrate different sensitivity levels for each of the four designs at different voltages Δ*v*_*c*_ (Δ*v*_*c*_ = Δ*v*_1_ = Δ*v*_2_), applied according to the wiring scheme depicted in Figs. 4, 6b, c. The measurement involved differentiating the output measured at each angle *θ* from the output at *θ* = 0, where no gravitational force is applied in the sensing direction. To mitigate the effects of time drift, two subsequent output measurements were taken for each angle.

Three different values of Δ*v*_*c*_ were applied, resulting in the curves shown in Fig. 7a–d: zero voltage, mid-voltage, and high-voltage. The zero-voltage corresponds to the normal case where the spring stiffness is not altered. The high-voltage corresponds to the highest voltage Δ*v*_*c*_ that we could apply before reaching bistability (negative stiffness at the as-fabricated position) or unstable oscillating output. The sensitivity at the high voltage is the highest sensitivity that we could practically reach using the measurement setup. The mid-voltage corresponds to a voltage in-between where the sensitivity is similarly in-between the lowest and highest sensitivity. The applied voltages Δ*v*_*c*_ and the gain in sensitivity compared to the initial sensitivity (zero-voltage) are shown in Table 2.

**Table 2:** Dimensions for the opposite inclined beam springs implemented in the fabricated accelerometer

	mid-voltage			high-voltage	
Accelerometers	voltage (V)	sensitivity gain		voltage (V)	sensitivity gain
Spring 1	2.6	2.07		2.95	4.34
Spring 2	4.55	7.34		4.76	12.62
Spring 3	3.5	2.81		3.8	6.64
Spring 4	3.1	3.61		3.4	7.1
Spring 5	-	-		3.195	55.85

Additional characteristic curves and data are illustrated in Fig. 7e and Table 2 for accelerometer design 5 as it experimentally reached the highest gain in sensitivity. A sensitivity gain of more than 55 times (calculated in the linear range) was reached at Δ*v*_*c*_ = 3.195 V. The variation in sensitivity becomes very sharp just before the negative-stiffness voltage, and the determination of the high voltage that we could apply is very accurate.

The measurements in Fig. 7e show a nonlinear relationship between the acceleration and accelerometer output at high voltages (Δ*v*_*c*_ > 3). This is probably because of the nonlinear relationship between the proof mass displacement and capacitance variation in the sensing electrodes. At high voltage (Δ*v*_*c*_), the spring stiffness becomes very low, the proof mass displacement becomes quite high compared to the initial gap between comb drives, and the linear output of the accelerometer becomes limited to a smaller amplitude of acceleration. Besides, a non-symmetrical behavior (non-equivalent outputs for positive and negative acceleration) can also be noticed in the sensitivity curves at high voltages. This is mainly because of the difference in wiring and contact resistance between the four sides of the spring mechanism and the sharp variation in sensitivity for a small variation in Δ*v*_1_ or Δ*v*_2_ near the critical voltage. A symmetrical behavior can be obtained by slightly and accurately varying Δ*v*_1_ or Δ*v*_2_ (Δ*v*_1_ ≠ Δ*v*_2_) to compensate for the difference in resistance between the four sides of the spring mechanism.

Figure 7f reveals a very good agreement in the comparison between the measured sensitivity in sensor 5 and the calculated sensitivity based on the analytical equations in the previous section. The total sensitivity is defined as the output-to-input ratio of the sensor, where the output is in mV and the input is proportional to the gravitational acceleration. In the analytical calculation, we consider that the total sensitivity is inversely proportional to the varying spring stiffness (14) and is multiplied by a constant which is determined by comparison to the measurements. The varying spring stiffness is calculated from (12), considering the inclined beam dimensions in the spring mechanism (Table 1) and the silicon material properties (Table 3). Note that the actual electrical resistivity of Silicon might be slightly different (it depends on doping concentration and temperature) due to the additional wiring and contact resistance added to the circuit.

**Table 3:** Silicon material properties considered in the calculation for the sensitivity curve in Fig. 7f

Material properties	Value
Expansion coefficient *α*	Thermally dependent^54^
Young’s modulus *E* (GPa)	169
Electrical resistivity *ρ*_0_ (Ωcm)	0.015135
Thermal conductivity *K*_*p*_ (W/mK)	149

**Fig. 8:**
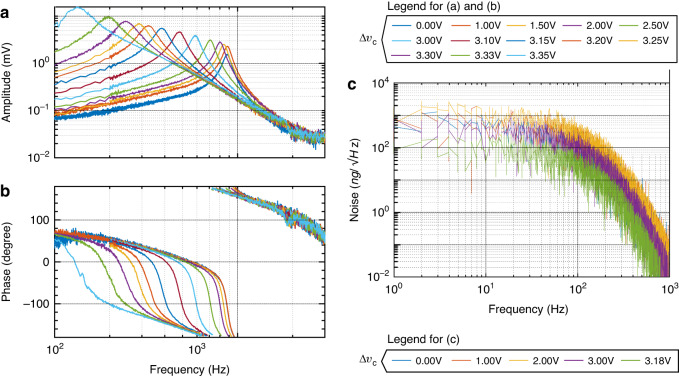
Frequency and noise response. **a** Frequency response for sensor 5 obtained with different applied voltages with 1.5 V DC and 0.5 V AC. The curves showcase the impact of different voltages (Δ*v*_*c*_) applied to the inclined beams to achieve different stiffness and resonance frequency levels. **b** Phase shift response for sensor 5. **c** Noise floor of sensor 5 with and without stiffness tuning

The resonant frequency is another measurable parameter that changes with the stiffness of the spring (13). The frequency response for accelerometer design 5 is measured with a lock-in amplifier, where a 1.5 V DC voltage is combined with a 0.5 V AC voltage applied to the actuating comb drives. As shown in Fig. 8a, b, the measurements showed an increase in amplitude and a reduction in the resonant frequency when the applied voltage Δ*v*_*c*_ is closer to the negative-stiffness voltage. This indicates a reduction in spring stiffness and demonstrates the effective functionality of the tunable spring mechanism.

The noise floor curves of accelerometer design 5 under various actuation voltages Δ*v*_*c*_ are depicted in Fig. 8c. The noise floor represents the baseline level of noise in the accelerometer system and is a crucial indicator of its resolution. In Table 4, the corresponding noise floor limits for the same sensor within the frequency range of 10-1000 Hz are provided. The noise floor is measured in units of ng/$\sqrt{{{{\rm{Hz}}}}}$, where “ng" denotes nanogravity, and Hz represents the frequency over which the noise is calculated. Notably, the noise floor is significantly reduced to 69 ng/$\sqrt{{{{\rm{Hz}}}}}$ when Δ*v*_*c*_ approaches the zero stiffness limit. This reduction is 6.52 times smaller than that observed without stiffness tuning, resulting in a noise floor of 450 ng/$\sqrt{{{{\rm{Hz}}}}}$.

**Table 4:** Noise floor of accelerometer design 5 obtained at the different actuation voltages Δ*v*_*c*_ in Fig. 4

Actuation voltage Δ*v*_*s*_ (V)	Noise floor (ng/$\sqrt{{{{\rm{Hz}}}}}$, @10-1000 Hz)
0	<450
1	<550
2	<525
3	<252
3.18	<69

The measurements results show a slight increase in noise floor for low voltages (Δ*v*_*c*_ = 1 V and 2 V), which can be attributed to the temperature rise resulting from electroheated springs. It is worth noting that this temperature increase is likely localized to the spring area, with limited impact on the temperature of the proof mass, where the primary source of Brownian noise occurs. Thus, with the further increase of the actuation voltage, the sensitivity and resolution of the MEMS accelerometer dramatically increase due to the reduction in stiffness.

## Discussion

The experimental measurements prove the functionality of the proposed tunable stiffness mechanism and its efficiency in tuning and significantly increasing the sensitivity of accelerometers. The experiments showed a significant increase in the sensitivity of sensor design 5 and a relatively lower increase for the other designs. The theory of the tunable stiffness mechanism showed very good agreement with the experimental measurements. In theory, there is no limit to the sensitivity that can be reached as long as we can provide stable and highly accurate powering for the tunable stiffness mechanism. Thus, by optimizing the geometry and measurement conditions, there is a high potential to achieve much higher sensitivity and unprecedented performance for micromachined accelerometers.

The geometry optimization for the spring mechanism is not limited to the dimensions of inclined beams. Other beam shapes in the spring mechanism, such as curved, arc-shaped, Z-shaped, and kink-shaped, can be investigated^26,36^. These shapes for the spring beams should enable negative stiffness behavior, a condition necessary to achieve near-zero stiffness behavior and thus maximize sensitivity. In this paper, we chose to consider the inclined shape for its simple structure and ease of fabrication.

Regarding the dimensions, the length (l), thickness (t), mid-height (h), and width (b) define the geometry of the inclined beam (Fig. 1a). The width is practically the same width as the device layer in the fabricated prototypes. The other dimensions can be determined respecting fabrication constraints and to avoid exceeding stress limits after deformation^35,45^. Note that these dimensions determine the stiffness and force-displacement characteristics at rest and the electrothermal voltages required to reach near-zero stiffness behavior. Particularly in the accelerometer application, these dimensions can be chosen to improve the gain in sensitivity (18) and its variation with respect to applied electrothermal voltages (19).

The gain in sensitivity, in theory, reduces with the height-to-thickness ratio *Q* and increases as the normalized electrothermal voltage *V*_*c*_ approaches *V*_0_ (where the stiffness is at a zero level). The gain in sensitivity for inclined beams is calculated as follows:

18 $${G}_{S}=\frac{{S}_{(V = {V}_{c})}}{{S}_{(V = 0)}}=\frac{{k}_{{c}_{0}(V = 0)}}{{k}_{{c}_{0}(V = {V}_{c})}}=\frac{192({Q}^{2}+1)}{192{Q}^{2}-\frac{1}{{{{\Lambda }}}_{e}({V}_{c})}}$$

The experimental setup noise and the limited resolution of the powering devices do not allow *V*_*c*_ (Δ*v*_*c*_) to precisely reach *V*_0_ (Δ*v*_0_), where the sensitivity theoretically approaches infinity. To achieve an increased sensitivity gain in practice, it is necessary to enhance the ratio of the sensitivity gain increment to the increment in applied current/voltage (∂*G*_*S*_/∂Δ*v*_*c*_). A higher ratio ∂*G*_*S*_/∂Δ*v*_*c*_ enables a higher gain in sensitivity when the minimum voltage increment (∂Δ*v*_*c*_) is added to Δ*v*_*c*_. Increasing this ratio ensures the attainment of higher sensitivity gains as Δ*v*_*c*_ approaches Δ*v*_0_, while staying within the positive stiffness limit. Note that ∂Δ*v*_*c*_ depends on the quality of the measurement setup. The analytical expression for ∂*G*_*S*_/∂Δ*v*_*c*_ considering inclined beams is provided below:

19 $$\frac{\partial {G}_{S}}{\partial {{\Delta }}{v}_{c}}=\sqrt{\frac{\alpha }{{\rho }_{0}{K}_{p}}}\frac{l}{t}\frac{768({Q}^{2}+1){V}_{c}{{{\Lambda }}}_{a}^{2}({V}_{c})}{{\left(192{Q}^{2}{{{\Lambda }}}_{e}({V}_{c})-1\right)}^{2}}$$

The gain variation in (19) increases with the length-to-thickness ratio (*l*/*t*), which is a possible explanation for why we reached a higher gain in sensitivity with spring design 5. For the accelerometers with small-length springs (accelerometers 1-3), within a certain range of Δ*v*_*c*_ below Δ*v*_0_, we noticed an oscillatory output from the accelerometers in the experiments without applying any external acceleration. We observed a higher sensitivity to acceleration in this range of Δ*v*_*c*_, but the output signal is combined with an oscillatory signal. This phenomenon needs further investigation to understand its origin. Some possible explanations for the oscillatory output are that the resonant mode was triggered for a certain reason or the output is affected by fluctuations from the powering devices. This phenomenon limited the possibility of further increasing the sensitivity gain in these devices.

The sensitivity gain is higher in sensor 2 compared to sensor 3, which is in agreement with equation (19) suggesting that the gain variation with voltage is higher for lower values of *Q*. However, in the devices with straight-beam spring mechanism (accelerometers 1 and 4 have *h* = 0), tuning the sensitivity near zero stiffness was more challenging as the spring beams tend to buckle in one direction, and their displacement cannot be controlled in the two directions as is the case with inclined beams, which can be controlled to move in both directions of curvature. Thus, it is recommended to reduce the value of the height-to-thickness ratio *Q* in the design while keeping the height *h* larger than a minimum value. This minimum value enables the control of the spring mechanism displacement by changing Δ*v*_1_ and Δ*v*_2_.

Symmetric sensitivity is a requirement for the good quality of the accelerometer. To ensure symmetric sensitivity, Δ*v*_1_ and Δ*v*_2_ should be accurately calibrated to compensate for the different wiring resistance on the four sides of the spring mechanism. Practically, symmetric sensitivity can be calibrated by placing the accelerometer at rest on a stable support in a horizontal position (*θ* = 0), where no gravitational or inertia forces are applied. Set a certain value for Δ*v*_1_ = Δ*v*_2_, then slightly change Δ*v*_1_ or Δ*v*_2_ to reach the same output before electroheating. The latter indicates that the proof mass is at its as-fabricated position and both sides of the spring mechanism are similarly electroheated. While this adjustment process is done manually in the presented experiments, an automated adjustment system for the input voltages would enhance the efficiency and repeatability of the tuning process and could further improve the accuracy and control of the near-zero stiffness point.

Compared to traditional accelerometers, the proposed mechanism requires additional power consumption to electro-heat the spring. However, the electrical power required for heating the springs was limited to only a few milliwatts in the case of the experimental devices, which is relatively acceptable for general applications. An on-off tuning scheme can be employed to reduce power consumption if continuous measurement is not necessary. From another perspective, it’s important to note that the heating time should not have an impact on the accelerometer’s performance. This is because electroheating for microbeams is quite fast^46^, and the measurements assume a stable stiffness once the tuning process is completed and the temperature conditions within the spring structure have stabilized.

The proposed mechanism also poses some other concerns, such as the reduction in dynamic range and limited bandwidth (due to the shift in resonance frequency). The largest acceleration amplitude that can be measured is limited by the proof mass displacement, which is inversely proportional to the stiffness (*ma* = *kd*). However, the minimum measurable resolution of acceleration is limited by the noise floor, which reduces after reducing the stiffness but with a smaller proportion (as shown in the example of accelerometer design 5). This non-proportionality indicates a reduction in dynamic range at small stiffness levels. Another related concern is the nonlinear output of the accelerometer for a large displacement of the proof mass.

The bandwidth of the accelerometer is limited by the resonance frequency. As demonstrated in the experiments (Fig. 8a), the resonance frequency decreases with stiffness according to the laws of physics. By enhancing sensitivity through this approach, the accelerometer becomes well-suited for scenarios where high precision and sensitivity in acceleration measurements are of paramount importance. In fields like seismology^47^, gravimetry^48^, and vibration monitoring^49^, where low-frequency and low-amplitude signals are prevalent, the ability to detect and accurately measure such signals is crucial. Furthermore, active tuning of the resonance frequency is advantageous for applications that leverage resonance amplification for small inputs, such as resonating micromirrors^50^ and gyroscopes^51^.

For applications requiring high precision along with improved linearity, dynamic range, and bandwidth, the designed accelerometer can address these demanding requirements by applying closed-loop techniques, which have already been shown to be quite effective for conventional MEMS accelerometers^52^. Applying closed-loop measurements with near-zero stiffness mechanisms is one of the main perspectives of this work, given its evident advantages.

Compared to the designs proposed in references^13,18,19,53^ (Table 5), our design exhibits a unique capability for tuning the sensitivity to compensate for fabrication tolerances and meeting diverse working bandwidth and sensitivity requirements. Furthermore, our design has a smaller proof mass weight and sensing capacitance compared to existing designs, resulting in lower sensitivity and limited resolution. It is important to note that different accelerometers utilize different sensing principles to measure the displacement of the proof mass under acceleration. As a result, the sensitivity unit varies. Also, it is worth mentioning that the weight of the proof mass in our work is only 1.5 *μ*g, whereas the proof mass weight in^19^ is 99.1 *μ*g. Furthermore, Middlemiss et al.^18^ and Tang et al.^13^ employ optical sensing for readout, which has a significantly higher scale factor than capacitive sensing. By increasing the weight of the proof mass, the sensing capacitance, or incorporating optical sensing into our design in future iterations, it is possible to further enhance both noise reduction and sensitivity.

**Table 5:** Comparison of Noise floor and Dynamic Range

Research group	Noise floor	Sensitivity
Boom and Kamp et al.^53^	2 $ng/\sqrt{Hz}$ (@ 28.1 Hz)	–
Middlemiss et al.^18^	8 $ng/\sqrt{Hz}$ (@ 1 Hz)	–
Zhang et al.^19^	51.8 $ng/\sqrt{Hz}$ (@ 1 Hz)	53 fF/g (3.78 mV/g)
Tang et al.^13^	8 $ng/\sqrt{Hz}$ (@ 1 Hz)	2200 kV/g
This work	69 $ng/\sqrt{Hz}$ (@ 50 Hz)	1 V/g

The external environment inevitably influences the resolution of accelerometers, particularly in highly accurate measurements. To mitigate this influence and ensure precision in real-world conditions, various strategies can be employed, such as encapsulating the accelerometer in sealed packages and integrating environmental sensors for adaptive sensitivity adjustments. The unique feature of active stiffness tuning enables the accelerometer to adapt to changing conditions through electrothermal adjustments. This active compensation for environmental disturbances enhances its robustness and suitability for applications requiring high precision in diverse environments.

Furthermore, the experimental setup and readout electronics can be optimized to achieve higher sensitivity and lower noise. Power sources with high resolution and stable output without fluctuations would help to more accurately approach the zero stiffness voltage and thus increase the sensitivity gain. Besides, more compact electronics developed specifically for the accelerometer with a tunable spring would help to simplify the wiring and reduce the electrical noise from the experimental setup. Performing the measurement in controlled environments and far from noise sources would enhance the sensitivity and resolution of the accelerometer.

## Conclusion

A tunable stiffness mechanism was implemented as a spring in micromachined accelerometers to tune and increase their sensitivity and improve performance. The spring mechanism consists of inclined beams connected to a middle rigid structure in parallel. The beams have a similar structure with opposite curvature and are electroheated at the same level to tune the spring stiffness and achieve near-zero stiffness. The accelerometer sensitivity increases by electrothermally reducing the spring stiffness to reach high sensitivity at near-zero stiffness. The theory of the tunable stiffness mechanism was first presented, and the governing equations were derived. The spring mechanism with different designs was implemented in an accelerometer with a known design and performance. The experimental measurements proved the successful functioning of the tunable spring concept and showed good agreement with the proposed theory. Certain prototypes achieved more than a 55-fold increase in sensitivity, while there is room for optimizing the geometry and improving the measurement setup and conditions to achieve even higher gains in sensitivity. The principle of the tunable spring mechanism can be generalized to many types of accelerometers and can be implemented with different types of transducers.

## Methods

### Electrical simulations

In the standard measurement, a periodic carrier signal Δ*v*_*ac*_ is applied to the proof mass through the spring structure. The readout circuit then amplifies the electric potential variation in the sensing electrodes, which is proportional to the capacitance variation. In this case, the same electric potential is applied across the springs and proof mass. However, with the novel spring mechanism, there is a difference where the spring beams should be electro-heated with a potential difference between their beam ends, as clarified in Fig. 4. This raises a concern about the functionality of the readout circuit if the electric potential gradually varies across the proof mass structure.

The spring beams have a thin structure and thus high electrical resistance compared to the effective resistance of the proof mass. Additionally, a symmetric potential difference is applied between both sides of the springs (i.e., + Δ*v*_1_ and + Δ*v*_2_ on one side, and − Δ*v*_1_ and − Δ*v*_2_ on the other side, as shown in Fig. 4). These conditions result in a near Δ*v*_*ac*_ potential across the proof mass structure, particularly in the middle area next to the sensing electrodes. Figure 3b presents the results of a finite element simulation using Ansys, showing the distribution of electric potential across the springs and proof mass structure after applying +0.5 V and − 0.5 V on the spring ends.

The simulation in Fig. 3b was performed using a structural-thermoelectric planar element (Plane 223) and considers the properties of silicon material. The simulation shows a relatively small variation in the potential difference in the middle zone of the proof mass next to the sensing electrodes. Considering the small variation in electric potential and its symmetric distribution, the electroheating should not affect the functioning of the readout circuit. This is also validated by experiments that demonstrated successful measurements with the electroheating.

### Fabrication and sample preparation

The fabrication was conducted separately in the cleanrooms of KAUST and KU Leuven, resulting in functional devices in both fabrication runs. The accelerometers were fabricated on Silicon On Insulator (SOI) wafers following the same process described in^24^. The device and handle layers of the wafer (25 *μm* and 300 *μm*, respectively) were etched using Deep Reactive Ion Etching, and the device parts were released with vapor HF. In the release phase, the proof mass was separated from a block in the handle layer, which improves the etching quality and reduces the risks of failure during the etching and release phases^24^.

After fabrication, a low-stress glue is applied to the four corners and cured to secure the accelerometer die on a carrier PCB. The pads on the die and carrier PCB are wire bonded and connected to the sensing/feedback circuit using header pins. Since the sensing/feedback circuit was previously developed for measuring from a standard differential capacitive accelerometer, an adapter circuit was prepared to apply different voltages to the four sides of the spring mechanism. The adapter circuit is connected between the sensing/feedback circuit and the carrier PCB.

### Experimental setup

In the wiring scheme, the spring mechanism is powered by an arbitrary signal generator (Rigol DG3061A) that generates a 1 MHz 2 Vpp AC signal (Δ*v*_*AC*_), and two power supplies (Keysight E36312A) that generate four DC outputs ($+\frac{1}{2}{{\Delta }}{v}_{1}$, $-\frac{1}{2}{{\Delta }}{v}_{1}$, $+\frac{1}{2}{{\Delta }}{v}_{2}$, and $-\frac{1}{2}{{\Delta }}{v}_{2}$). The AC signal is divided into four signals and then combined with the four DC signals applied to the spring mechanism using bias tees. The AC and combined DC + AC signals are transmitted using coaxial cables. A power supply (Delta Elektronika EST 150) provides ± 5 V DC inputs to power the sensing/feedback circuit.

The sensing circuit outputs an analog voltage proportional to the applied acceleration in the sensing direction of the accelerometer. The analog output is measured using readout devices, mainly a high-precision multimeter (Keithley DMM6500) or an oscilloscope (Rohde *&* Schwarz RTB2004). A lock-in amplifier (Zurich Instruments UHF) is used to measure the noise and frequency response. During these measurements, the actuating comb-drives are controlled by the amplifier to induce motion in the proof mass at different frequencies. It is important to note that the ground level is consistent across all devices, and the handle support in the accelerometer die is grounded for the purpose of shielding.

## References

[1] Wang M, Cui J, Huang Y, Wu W, Du X (2021). Schmidt st-ekf for autonomous land vehicle sins/odo/ldv integrated navigation. IEEE Trans. Instrum. Meas..

[2] Bao J, Li D, Qiao X, Rauschenbach T (2020). Integrated navigation for autonomous underwater vehicles in aquaculture: A review. Inf. Process. Agr..

[3] Zhang D, Zheng X, Xie Y, Hu X (2022). Angular-accelerometer-based flexible-state estimation and tracking controller design for hypersonic flight vehicle. Aerospace.

[4] Hou Y, Jiao R, Yu H (2021). Mems based geophones and seismometers. Sens. Actuators A: Phys..

[5] Santoli F (2020). Isa, a high sensitivity accelerometer in the interplanetary space. Space Sci. Rev..

[6] Liu Q (2021). High figure of merit and low cross sensitivity fiber bragg grating accelerometer based on double grid-diaphragms. IEEE Sens. J..

[7] Ibrahim A, Eltawil A, Na Y, El-Tawil S (2020). Accuracy limits of embedded smart device accelerometer sensors. IEEE Trans. Instrum. Meas..

[8] Bista S, Debache I, Chaix B (2020). Physical activity and sedentary behaviour related to transport activity assessed with multiple body-worn accelerometers: the record multisensor study. Public Health.

[9] Regterschot GRH, Selles RW, Ribbers GM, Bussmann JBJ (2021). Whole-body movements increase arm use outcomes of wrist-worn accelerometers in stroke patients. Sensors.

[10] Tsai J-M, Sun I-C, Chen K-S (2021). Realization and performance evaluation of a machine tool vibration monitoring module by multiple mems accelerometer integrations. Int. J. Adv. Manuf. Technol..

[11] Gupta N (2021). Characterization of soi mems capacitive accelerometer under varying acceleration shock pulse durations. Microsyst. Technol..

[12] Wang C (2020). Sensors.

[13] Tang S (2019). A high-sensitivity mems gravimeter with a large dynamic range. Microsyst. Nanoeng..

[14] Wu W (2018). A nano-g micromachined seismic sensor for levelling-free measurements. Sens. Actuators, A: Phys..

[15] Han C, Li C, Zhao Y, Li B (2021). High-stability quartz resonant accelerometer with micro-leverages. J. Microelectromech. Syst..

[16] Lanniel A, Boeser T, Alpert T, Ortmanns M (2021). Low-noise readout circuit for an automotive mems accelerometer. IEEE Open J. Solid-State Circuits Soc..

[17] Haub M, Bogner M, Guenther T, Zimmermann A, Sandmaier H (2021). Development and proof of concept of a miniaturized mems quantum tunneling accelerometer based on ptc tips by focused ion beam 3d nano-patterning. Sensors.

[18] Middlemiss RP (2016). Measurement of the earth tides with a mems gravimeter. Nature.

[19] Zhang H, Wei X, Ding Y, Jiang Z, Ren J (2019). A low noise capacitive mems accelerometer with anti-spring structure. Sens. Actuators A: Phys..

[20] Boom, B. et al. Nano-G accelerometer using geometric anti-springs. In *2017 IEEE 30th International Conference on Micro Electro Mechanical Systems (MEMS)* 33–36 (IEEE, 2017).

[21] Guo Y, Ma Z, Zhang T, Zheng X, Jin Z (2021). A stiffness-tunable mems accelerometer. J. Micromech. Microeng..

[22] Duan Y (2020). Design and numerical performance analysis of a microgravity accelerometer with quasi-zero stiffness. Smart Mater. Struct..

[23] Hussein H, Khan F, Younis MI (2020). A monolithic tunable symmetric bistable mechanism. Smart Mater. Struct..

[24] Sari I, Zeimpekis I, Kraft M (2012). A dicing free soi process for mems devices. Microelectron. Eng..

[25] Utz A (2018). A high-precision and high-bandwidth mems-based capacitive accelerometer. IEEE Sens. J..

[26] Hussein H, Younis MI (2020). Analytical Study of the Snap-Through and Bistability of Beams With Arbitrarily Initial Shape. J. Mechanisms Robot..

[27] Cao Y, Derakhshani M, Fang Y, Huang G, Cao C (2021). Bistable structures for advanced functional systems. Adv. Funct. Mater..

[28] Chen S (2022). Continuous carbon fiber reinforced composite negative stiffness mechanical metamaterial for recoverable energy absorption. Composite Struct..

[29] Wu W (2020). Measurement of tidal tilt by a micromechanical inertial sensor employing quasi-zero- stiffness mechanism. J. Microelectromechanical Syst..

[30] Smreczak M, Tissot-Daguette L, Thalmann E, Baur C, Henein S (2022). A load cell with adjustable stiffness and zero offset tuning dedicated to electrical micro- and nanoprobing. Precis. Eng..

[31] Iqbal S, Malik A (2019). A review on mems based micro displacement amplification mechanisms. Sens. Actuators A: Phys..

[32] Ghavidelnia N, Yin K, Cao B, Eberl C (2023). Curly beam with programmable bistability. Mater. Des..

[33] Gan J, Xu H, Zhang X, Ding H (2022). Design of a compliant adjustable constant-force gripper based on circular beams. Mechanism Mach. Theory.

[34] Hussein H, Fariborzi H (2022). Accurate sensorless multistable microsystem with a single actuator. Front. Mech. Eng..

[35] Hussein H, Younis MI, Fariborzi H (2020). Task feasibility of V shape electrothermal actuators. Eng. Res. Express.

[36] Hussein H, Fariborzi H, Younis M (2020). Modeling of beam electrothermal actuators. J. Microelectromechanical Syst..

[37] Qiu J, Lang J, Slocum A (2004). A Curved-Beam Bistable Mechanism. J. Microelectromechanical Syst..

[38] Hussein H, Le Moal P, Bourbon G, Haddab Y, Lutz P (2015). Modeling and Stress Analysis of a Pre-Shaped Curved Beam: Influence of High Modes of Buckling. Int. J. Appl. Mech..

[39] Hu B (2021). A novel trapezoidal scaln/aln-based mems piezoelectric accelerometer. IEEE Sens. J..

[40] Carlo MD, Leonardis FD, Soref RA, Passaro VMN (2021). Design of an exceptional-surface-enhanced silicon-on-insulator optical accelerometer. J. Lightwave Technol..

[41] Mukherjee R, Basu J, Mandal P, Guha PK (2017). A review of micromachined thermal accelerometers. J. Micromech. Microeng..

[42] Hou Y, Yuan W, Wang DF, Itoh T, Maeda R (2022). A low 1/f noise tunnel magnetoresistance accelerometer. IEEE Trans. Instrum. Meas..

[43] Qiao Y, Arabi M, Xu W, Zhang H, Abdel-Rahman EM (2021). The impact of thermal-noise on bifurcation mems sensors. Mech. Syst. Signal Process..

[44] Dutta S (2018). Effect of vacuum packaging on bandwidth of push-pull type capacitive accelerometer structure. Microsyst. Technol..

[45] Hussein H (2019). On the design of a preshaped curved beam bistable mechanism. Mech. Mach. Theory.

[46] Hussein H (2016). Dynamic electro-thermo-mechanical modelling of a u-shaped electro-thermal actuator. J. Micromech. Microeng..

[47] Qu Z (2022). 2.4 ng/*√*hz low-noise fiber-optic mems seismic accelerometer. Opt. Lett..

[48] Yang L (2022). A highly stable and sensitive mems-based gravimeter for long-term earth tides observations. IEEE Trans. Instrum. Meas..

[49] Zhang P (2022). A fiber-optic accelerometer based on extrinsic fabry-perot interference for low frequency micro-vibration measurement. IEEE Photonics J..

[50] Ruotsalainen K, Morits D, Ylivaara OME, Kyynäräinen J (2022). Resonating aln-thin film mems mirror with digital control. J. Optical Microsyst..

[51] Ranji AR (2022). Recent advances in mems-based 3d hemispherical resonator gyroscope (hrg)-a sensor of choice. Micromachines.

[52] Li X, Zheng Y, Kong X, Liu Y, Tang D (2020). Research on high-resolution miniaturized mems accelerometer interface asic. Sensors.

[53] Kamp, P. *Towards an ultra sensitive seismic accelerometer*. Master’s thesis, University of Twente (2016).

[54] Okada Y, Tokumaru Y (1984). Precise determination of lattice parameter and thermal expansion coefficient of silicon between 300 and 1500 K. J. Appl. Phys..

